# The phylogenetic position of *Anacanthorus* (Monogenea, Dactylogyridae) parasitizing Brazilian serrasalmids (Characiformes)

**DOI:** 10.1051/parasite/2019045

**Published:** 2019-07-23

**Authors:** Juliana Moreira, José L. Luque, Andrea Šimková

**Affiliations:** 1 Curso de Pós-Graduação em Biologia Animal da Universidade Federal Rural do Rio de Janeiro BR 465, Km 7, Caixa Postal 74.540 23890-000 Seropédica RJ Brazil; 2 Departamento de Parasitologia Animal, Universidade Federal Rural do Rio de Janeiro Caixa Postal 74.540 23851-970 Seropédica RJ Brazil; 3 Department of Botany and Zoology, Faculty of Science, Masaryk University Kotlářská 2 Brno 611 37 Czech Republic

**Keywords:** *Anacanthorus*, Dactylogyridae, Serrasalmidae, Neotropical region, 28S rDNA, Molecular phylogeny

## Abstract

*Anacanthorus* (Anacanthorinae) is one of the most speciose and common genera of neotropical monogeneans, yet there are still many gaps in our knowledge concerning their diversity and phylogeny. We performed phylogenetic analyses of molecular sequences in order to investigate the phylogenetic position within the Dactylogyridae of *Anacanthorus* spp. infesting serrasalmids from two Brazilian river basins. Sequences of partial 28S rDNA obtained for nine species of *Anacanthorus* and *Mymarothecium viatorum* parasitizing serrasalmids and the published sequences of other members of the Dactylogyridae were included in the phylogenetic reconstruction. Phylogenetic analyses supported the monophyly of anacanthorine monogeneans. The Anacanthorinae (represented in this study by *Anacanthorus* spp.) formed a monophyletic group included in a large clade together with a group of solely freshwater Ancyrocephalinae and species of the Ancylodiscoidinae. *Mymarothecium viatorum* (Ancyrocephalinae) was placed within the clade of freshwater Ancyrocephalinae. The phylogenetic analyses indicated that the relationships among species of *Anacanthorus* reflect those of their serrasalmid hosts: the first subgroup includes a species specific to hosts assigned to *Piaractus*, a member of the “pacus” lineage; the second subgroup includes a species parasitizing the “*Myleus*-like pacus” lineage; and the third subgroup includes species parasitizing the lineage of the “true piranhas”. We suggest that *Anacanthorus* and their serrasalmid hosts can be considered a useful model to assess host-parasite biogeography and coevolution in the neotropics. However, future studies focusing on a wider spectrum of host species and their specific *Anacanthorus* spp. are needed in order to investigate coevolution in this highly diversified system.

## Abstract (fr)

*Anacanthorus* (Anacanthorinae) est parmi les genres les plus riches en espèces et les plus communs parmi les monogènes néotropicaux. Cependant, nos connaissances sur sa diversité et sa phylogénie sont encore lacunaires. Nous avons effectué des analyses phylogénétiques de séquences moléculaires afin d’étudier la position phylogénétique au sein des Dactylogyridae d’*Anacanthorus* spp. infestant des Serrasalmidés de deux bassins fluviaux brésiliens. Les séquences d’ADNr 28S partiel obtenues pour 9 espèces d’*Anacanthorus* et *Mymarothecium viatorum* parasitant les Serrasalmidés et les séquences publiées d’autres membres des Dactylogyridae ont été incluses dans la reconstruction phylogénétique. Les analyses phylogénétiques ont confirmé la monophylie des Anacanthorinae. Les Anacanthorinae (représentés dans cette étude par *Anacanthorus* spp.) forment un groupe monophylétique inclus dans un grand clade avec un groupe d’Ancyrocephalinae uniquement d’eau douce et d’espèces d’Ancylodiscoidinae. *Mymarothecium viatorum* (Ancyrocephalinae) a été placé dans le clade des Ancyrocephalinae d’eau douce. Les analyses phylogénétiques indiquent que les relations entre les espèces d’*Anacanthorus* reflètent celles de leurs hôtes Serrasalmidés : le premier sous-groupe comprend une espèce spécifique aux hôtes attribués à *Piaractus*, un membre de la lignée « pacus » ; le deuxième sous-groupe comprend une espèce parasitant la lignée « pacus proche de *Myleus* » ; et le troisième sous-groupe comprend les espèces parasitant la lignée des « vrais piranhas ». Nous pensons qu’*Anacanthorus* et ses hôtes Serrasalmidés peuvent être considérés comme un modèle utile pour évaluer la biogéographie hôte-parasite et la coévolution dans les régions néotropicales. Cependant, de futures études portant sur un spectre plus large d’espèces hôtes et de leurs espèces spécifiques d’*Anacanthorus* spp. sont nécessaires pour étudier la coévolution dans ce système hautement diversifié.

## Introduction

Due to their direct life cycle, morphological adaptation, and high host specificity, gill monogeneans of fish are commonly studied parasites in the context of coevolution and biogeography of host-parasite systems [37, 58, 60]. The reconstruction of the evolutionary history of parasites and the investigation of their origin is the first step in coevolutionary studies. However, despite the enormous diversity of both freshwater fish and their monogenetic fauna (e.g., [1, 8, 14, 46]), coevolutionary studies of fish and their monogenean parasites from the Neotropical Region are still limited.

*Anacanthorus* Mizelle and Price, 1965 is one of the most diverse monogenean genera living on fish in the Neotropical Region. Of the 15 genera parasitizing serrasalmids, *Anacanthorus* currently comprises 75 nominal species, which are distributed on species of Bryconidae (15 species), Characidae (22), and Serrasalmidae (38) [9, 33, 42]. However, undescribed species of *Anacanthorus* have also recently been recorded on species of Erythrinidae [19, 20]. *Anacanthorus* belong to Anacanthorinae Price, 1967, which is restricted to the Neotropical Region, and at present this group accommodates only *Anacanthorus* and *Anacanthoroides* Kritsky & Thatcher, 1974, the latter being represented by only two species recorded on the Prochilodontidae.

The freshwater fish of the Serrasalmidae, representing the most common host group for *Anacanthorus*, include piranhas, pacus, and their relatives, and currently comprise 98 valid species distributed throughout South America [16]. Several species of this fish group are economically important for commercial fishing and aquaculture, especially in the Amazon region [3, 25, 36]. Many phylogenetic studies based on different molecular markers (e.g., mtDNA control region, 12S and 16S rRNA) have suggested that the Serrasalmidae form three major phylogenetic lineages, i.e., the “pacu” lineage (including *Colossoma*, *Mylossoma* and *Piaractus*), the “*Myleus*-like pacus” lineage (including *Mylesinus*, *Myleus*, *Ossubtus* and *Tometes*), and the “true piranhas” lineage (including *Catoprion*, *Metynnis*, *Pristobrycon*, *Pygocentrus*, *Pygopristis* and *Serrasalmus*) [47, 48, 64]. Serrasalmid fish exhibit enormous monogenean diversity. So far, 92 monogenean species belonging to 15 genera have been recorded on these fish. Most of these records originated from Brazil during the 1990s, when at least 8 genera and 61 species of monogeneans were described from piranhas and their relatives [8].

According to morphological analyses carried out by Kritsky and Boeger [28], the Anacanthorinae seem to represent a monophyletic group within the Dactylogyridae. Van Every and Kritsky [65] used the morphological characters of the haptoral hooks and reproductive organs to infer phylogenetic relationships between species of *Anacanthorus* from the “true piranhas” from the central Amazon. They suggested that this host-parasite system is a suitable model for studying biogeography and coevolution in the neotropics, although there are still many gaps in our knowledge concerning their diversity and phylogeny (i.e., the phylogenetic position of *Anacanthorus* within the Dactylogyridae and interspecific relationships within the genus).

Using the complete SSU (18S rDNA), Müller et al. [45] performed a study on *Anacanthorus penilabiatus* Boeger, Husak & Martins 1995 [6] and *Mymarothecium viatorum* Boeger, Piasecki & Sobecka, 2002 [7] (Ancyrocephalinae), both parasites of the pacu *Piaractus mesopotamicus* (Holmberg, 1887), focusing on the phylogenetic position of these monogeneans within the Dactylogyridae. Recently, Graça et al. [20] investigated the coevolutionary processes between selected species of *Anacanthorus* and their hosts in southern Brazil, and identified host-parasite cospeciation at the level of host families (Serrasalmidae, Bryconidae and Erythrinidae) and their specific *Anacanthorus* spp.

Considering the richness of *Anacanthorus* (the highest of all genera parasitizing Characiformes in the neotropics), the high host specificity exhibited by *Anacanthorus* species, and the scarcity of phylogenetic studies focused on these dactylogyrids, the aim of this study was to investigate the phylogenetic position of *Anacanthorus* spp. within the Dactylogyridae that infest serrasalmids from two Brazilian river basins based on the analysis of partial 28S rDNA sequences.

## Materials and methods

### Specimen collection and processing

Fish were caught by local fishermen with gill nets and hooks from the following localities in Brazil: the Miranda River (20°11′27″S; 56°30′19″W), the Negro River (Mato Grosso do Sul) (19°34′40″S; 56°09′08″W), the Upper Paraná River (20°45′S; 53°16′W), and the Xingu River (3°12′S, 52°12′W) (see Table 1). Fish were examined for monogeneans immediately after capture. All experimental handling was carried out in compliance with animal safety and ethics rules issued by the Federal Rural University of Rio de Janeiro (UFRRJ). Gills excised from fish were placed in Petri dishes with tap water and examined for monogeneans using a dissecting microscope. Parasites were placed individually in a drop of water on a slide and the haptor of each specimen was excised from the body and preserved in absolute ethanol for molecular analyses. The rest of the body was mounted in a mixture of glycerine and ammonium picrate (GAP) and kept as a molecular voucher. Additionally, some entire specimens were mounted in GAP and kept as paragenophore specimens (see Astrin et al. [4] for terminology). Species determinations were mainly based on the morphology of the male copulatory organ and of the haptoral hooks following the original descriptions by Boeger and Kritsky [5], Van Every and Kritsky [65], and Boeger et al. [6]. After morphological evaluation, specimens fixed in GAP were remounted in Canada balsam according to the procedure described by Ergens [13]. Voucher specimens were deposited in the Helminthological Collection of the Institute Oswaldo Cruz (CHIOC), Rio de Janeiro, Brazil, under the catalogue numbers 40046 a–b and 40047 and in the Helminthological Collection of the Institute of Parasitology of the Czech Academy of Sciences, (IPCAS), Czech Republic, under the catalogue numbers M-702 – M-710.

**Table 1:** Species included in the phylogenetic analyses.

Parasite species	Host species	Host family	Locality	Accession number
Dactylogyridea				
Dactylogyridae				
*Actinocleidus recurvatus* Mizelle and Donahue, 1944 [41]	*Lepomis gibbosus* (Linnaeus)	Centrarchidae	River Dunaj, SR	AJ969951
*Aliatrema cribbi* Plaisance & Kritsky, 2004 [50]**	*Chaetodon citrinellus* (Cuvier, 1831)	Chaetodontidae	French Polynesia	AY820612
*Ameloblastella chavarriai* (Price, 1938) [53]	*Rhamdia quelen* (Quoy & Gaimard, 1824)	Heptapteridae	Lake Catemaco, MX	KP056251
*Ameloblastella* sp. 16 (from Mendoza-Palmero et al. [39])	*Hypophthalmus edentatus* Spix & Agassiz, 1829	Hypophtalmidae	River Nanay, PE	KP056255
*Ancyrocephalus paradoxus* Creplin, 1839 [10]	*Sander lucioperca* (Linnaeus)	Percidae	River Morava, CR	AJ969952
*Ancyrocephalus percae* (Ergens, 1966) [12]	*Perca fluviatilis* (Linnaeus)	Percidae	Lake Constance, GE	KF499080
***Anacanthorus amazonicus* Van Every & Kritsky, 1992 [** **65** **]**	*Serrasalmus maculatus* Kner, 1858	Serrasalmidae	River Negro, BR	MH843721
***Anacanthorus jegui* Van Every & Kritsky, 1992 [** **65** **]**	*Serrasalmus maculatus* Kner, 1858	Serrasalmidae	River Negro, BR	MH843720
***Anacanthorus lepyrophallus* Kritsky, Boeger, and Van Every, 1992 [** **29** **]**	*Serrasalmus maculatus* Kner, 1858	Serrasalmidae	River Baia, BR	MH843718
***Anacanthorus maltai* Boeger & Kritsky, 1988 [** **5** **]**	*Pygocentrus nattereri* Kner, 1858	Serrasalmidae	River Miranda, BR	MH843716
***Anacanthorus paraxaniophallus* Moreira, Carneiro, Ruz & Luque, 2019 [** **42** **]**	*Serrasalmus marginatus* Valenciennes, 1837	Serrasalmidae	River Paraná, BR	MH843717
***Anacanthorus penilabiatus* Boeger, Husak & Martins, 1995 [** **6** **]**	*Piaractus mesopotamicus* (Holmberg, 1887)	Serrasalmidae	River Paraná, BR	MH843719
***Anacanthorus rondonensis* Boeger & Kritsky, 1988 [** **5** **]**	*Pygocentrus nattereri* Kner, 1858	Serrasalmidae	River Miranda, BR	MH843714
***Anacanthorus thatcheri* Boeger & Kritsky, 1988[** **5** **]**	*Pygocentrus nattereri* Kner, 1858	Serrasalmidae	River Miranda, BR	MH843715
***Anacanthorus* sp. 1**	*Myleus setiger* Müller & Troschel, 1844	Serrasalmidae	River Xingu, BR	MH843722
*Bravohollisia roseta* Lim, 1995 [34]	*Pomadasys maculatus* (Bloch, 1793)	Haemulidae	Guangdong, CH	DQ537364
*Bychowskyella pseudobagri* Akhmerow, 1952 [2]	*Tachysurus fulvidraco* (Richardson, 1846)	Bagridae	Guangdong, CH	EF100541
*Dactylogyrus extensus* Mueller and Van Cleave, 1932 [44]	*Cyprinus carpio* (Linnaeus)	Cyprinidae	River Morava, CR	AJ969944
*Dactylogyrus inversus* (Goto and Kikuchi, 1917) [18]	*Lateolabrax japonicus* (Cuvier, 1828)	Lateolabracidae	CH	AY548928
*Euryhaliotrema perezponcei* García-Vargas, Fajer-Ávila & Lamothe-Argumedo, 2008 [17]	*Lutjanus guttatus* (Steindachner, 1869)	Lutjanidae	Bay Cerritos, MX	HQ615996
*Euryhaliotrematoides annulocirrus* (Yamaguti, 1968) [70]**	*Chaetodon vagabundus* (Linnaeus)	Chaetodontidae	AUT	AY820613
*Euryhaliotrematoides microphallus* (Yamaguti, 1968)[70]**	*Heniochus chrysostomus* Cuvier, 1831	Chaetodontidae	Palau	AY820617
*Haliotrema angelopterum* Plaisance, Bouamer & Morand, 2004 [49]	*Chaetodon kleinii* Bloch, 1790	Chaetodontidae	Palau	AY820620
*Haliotrema aurigae* (Yamaguti, 1968) [70]	*Chaetodon auriga* Forsskål, 1775	Chaetodontidae	AUT	AY820621
*Haliotrematoides guttati* García-Vargas, Fajer-Ávila & Lamothe-Argumedo, 2008 [17]	*Lutjanus guttatus* (Steindachner, 1869)	Lutjanidae	Bay Cerritos, MX	HQ615993
*Haliotrematoides spinatus* Kritsky & Mendoza-Franco in Kritsky, Yang & Sun, 2009 [32]	*Lutjanus guttatus* (Steindachner, 1869)	Lutjanidae	Pacific Coast, MX	KC663679
*Ligictaluridus pricei* (Mueller, 1936) [43]	*Ameiurus nebulosus* (Lesueur, 1819)	Ictaluridae	River Moldau, CR	AJ969939
* Mymarothecium viatorum* Boeger, Piasecki and Sobecka, 2002 [7]	*Piaractus mesopotamicus* (Holmberg, 1887)	Serrasalmidae	River Paraná, BR	MH843723
*Onchocleidus similis* (Mueller, 1936) [43]	*Lepomis gibbosus* (Linnaeus)	Centrarchidae	River Danube, SR	AJ969938
*Onchocleidus* sp.	*Lepomis macrochirus* Rafinesque, 1819	Centrarchidae	Guangzhou, CH	AY841873
*Parasciadicleithrum octofasciatum* Mendoza-Palmero, Blasco-Costa, Hernández-Mena & Pérez-Ponce de León, 2017 [40]	*Rocio octofasciata* (Regan, 1903)	Cichlidae	Unnamed creek in Ejido Reforma Agraria, MX	KY305885
*Pseudodactylogyrus anguillae* (Yin & Sproston, 1948) [71]	*Anguilla anguilla* (Linnaeus)	Anguillidae	River Dunaj, SR	AJ969950
*Pseudodactylogyrus bini* (Kikuchi, 1929) [26]	*Anguilla anguilla* (Linnaeus)	Anguillidae	Neusiedler Lake, AUS	AJ969949
*Pseudohaliotrema sphincteroporus* Yamaguti, 1953 [69]	*Siganus doliatus* Guérin-Méneville, 1829	Siganidae	Green Island, AUT	AF382058
*Quadriacanthus kobiensis* Ha Ky, 1968 [22]	*Clarias batrachus* (Linnaeus)	Clariidae	Guangzhou, CH	AY841874
*Sciadicleithrum meekii* Mendoza-Franco, Scholz & Vidal-Martínez, 1997 [38]	*Thorichthys meeki* Brind, 1918	Cichlidae	Unnamed creek in Ejido Reforma Agraria, MX	KY305889
*Sciadicleithrum splendidae* Kritsky, Vidal‐Martínez & Rodríguez‐Canul, 1994 [31]	*Parachromis friedrichsthalii* (Heckel, 1840)	Cichlidae	Laguna El Vapor, MX	KY305890
*Tetrancistrum* sp.	*Siganus fuscescens* (Houttuyn, 1782)	Siganidae	Heron Island, AUT	AF026114
*Thaparocleidus asoti* (Yamaguti, 1937) [68]	*Parasilurus asotus* (Linnaeus)	Siluridae	Chongqing City, CH	DQ157669
*Thaparocleidus siluri* (Zandt, 1924) [73]	*Silurus glanis* (Linnaeus)	Siluridae	River Morava, CR	AJ969940
*Unibarra paranoplatensis* Suriano & Incorvaia, 1995 [61]	*Aguarunichthys torosus* Stewart, 1986	Pimelodidae	Santa Clara, PE	KP056219
*Vancleaveus janauacaensis* Kritsky, Thatcher and Boeger, 1986 [30]	*Pterodoras granulosus* (Valenciennes, 1821)	Doradidae	River Itaya, PE	KP056240
Pseudomurraytrematidae				
*Pseudomurraytrema* sp.*	*Catostomus ardens* Jordan & Gilbert, 1881	Catostomidae	Snake River, Idaho	AF382059
Tetraonchinea				
Anoplodiscidae				
*Anoplodiscus cirrusspiralis* Roubal, Armitage & Rohde, 1983 [56]*	*Sparus aurata* (Linnaeus)	Sparidae	Sydney, AUT	AF382060
Tetraonchidae				
*Tetraonchus monenteron* (Wagener, 1857) [67]*	*Esox lucius* (Linnaeus)	Esocidae	River Morava, CR	AJ969953
Monocotylidea				
*Calicotyle affinis* Scott, 1911 [57]*	*Chimaera monstrosa* (Linnaeus)	Chimaeridae	Norway	AF382061
*Clemacotyle australis* Young, 1967 [72]*	*Aetobatus narinari* (Euphrasen, 1790)	Myliobatidae	Heron Island, AUT	AF348350
*Decacotyle lymmae* Young, 1967 [72]*	*Aetobatus narinari* (Euphrasen, 1790)	Myliobatidae	Heron Island, AUT	AF348359
*Dendromonocotyle octodiscus* Hargis, 1955 [23]*	*Dasyatis americana* (Hildebrand & Schroeder, 1928)	Dasyatidae	Gulf of Mexico	AF348352

* Species used as outgroups.

** *Euryhaliotrematoides* and *Aliatrema* were placed in subjective synonymy with *Euryhaliotrema* [27].

Species sequenced in this study are shown in bold.

Abbreviations: AUS – Austria, AUT – Australia, BR – Brazil, CH – China, CR – Czech Republic, GE – Germany, MX – Mexico, PE – Peru, SR – Slovak Republic.

### DNA extraction, amplification, and sequencing

DNA extraction was carried out in 200 μl of a 5% suspension of Chelex™ in deionized water containing 2 μl proteinase K, followed by incubation at 56 °C for 3 h and boiling at 95 °C for 8 min. The partial 28S rRNA gene region (D1–D3) was amplified using primers C1 and D2 [24] or U178 and L1642 [35]. For the C1 and D2 primers, PCR reactions were performed in a final volume of 15 μl containing 1 × PCR buffer, 1.5 mM of MgCl_2_, 0.2 mM of dNTPs, 0.5 mM of each oligonucleotide primer, 1 U of Taq DNA polymerase (Fermentas), 6.6 mg/ml of BSA, and 5 μl of genomic DNA, using the following cycling parameters: denaturation at 94 °C for 2 min, followed by 39 cycles of 94 °C for 20 s, annealing at 58 °C for 30 s, and elongation at 72 °C for 1 min 30 s, with a final elongation at 72 °C for 10 min. For the second pair of primers, PCR reactions were performed in a final volume of 25 μl containing 1 × PCR buffer, 3 mM of MgCl_2_, 0.2 mM of dNTP’s, 0.5 mM of each oligonucleotide primer, 1 U of Platinum Taq DNA polymerase (Invitrogen), 0.4 mg/ml of BSA, and 2.5 μl of genomic DNA, using the cycling profile described in Mendoza-Palmero et al. [39]. The PCR products were checked on 1% agarose gel and purified using an ExoSAP-IT kit (Ecoli, Bratislava, Slovakia), following the manufacturer’s instructions. Purified products were directly sequenced using PCR primer pair C1–D2 or U178–L1642 and two additional internal primers (1200F and 1200R, see Lockyer et al. [35]) with a BigDye Terminator Cycle Sequencing kit (Applied Biosystems, Foster City, CA, USA). Sequencing was performed on an ABI 3130 Genetic Analyzer (Applied Biosystems).

Contiguous sequences were assembled in Geneious (Geneious ver. 9 created by Biomatters, available from http://www.geneious.com/) and deposited in the GenBank database under the accession numbers listed in Table 1.

### Phylogenetic analyses

Nine species of *Anacanthorus* and *Mymarothecium viatorum* (host species are shown in Table 1) were sequenced for the partial 28S rRNA gene and aligned with 35 species belonging to the Dactylogyridea and four species of the Monocotylidea retrieved from GenBank (see Table 1). Sequences were aligned with the CLUSTAL W algorithm [63] implemented in Geneious. Ambiguously aligned regions were removed from the alignment with GBlocks v. 0.91 [62], using less stringent selection. Phylogenetic analyses were performed using species of Monocotylidae, Tetraonchidae, Anoplodiscidae, and Pseudomurraytrematidae as outgroups (see Table 1 for species). The substitution model TVM + I + G (the transversion model including the proportion of invariable sites and a gamma distribution), selected by the jModelTest [52] using the Bayesian information criterion, was used for Maximum Likelihood (ML) and Bayesian Inference (BI) analyses. The search for the ML tree and bootstrap resampling with 1000 replications were performed using PHYML [21] implemented in Geneious. BI analyses were performed using MrBayes v. 3.2 [55], running four Monte Carlo Markov chains for 10^7^ generations, with trees sampled every 10^3^ generations and the first 1000 samples discarded as “burn in”. In order to check the convergence and to confirm that the effective sample size (ESS) of each parameter was adequate for providing reasonable estimates of the variance in model parameters (i.e., ESS values >200), Tracer v. 1.6 [54] was used.

## Results

**Figure 1:**
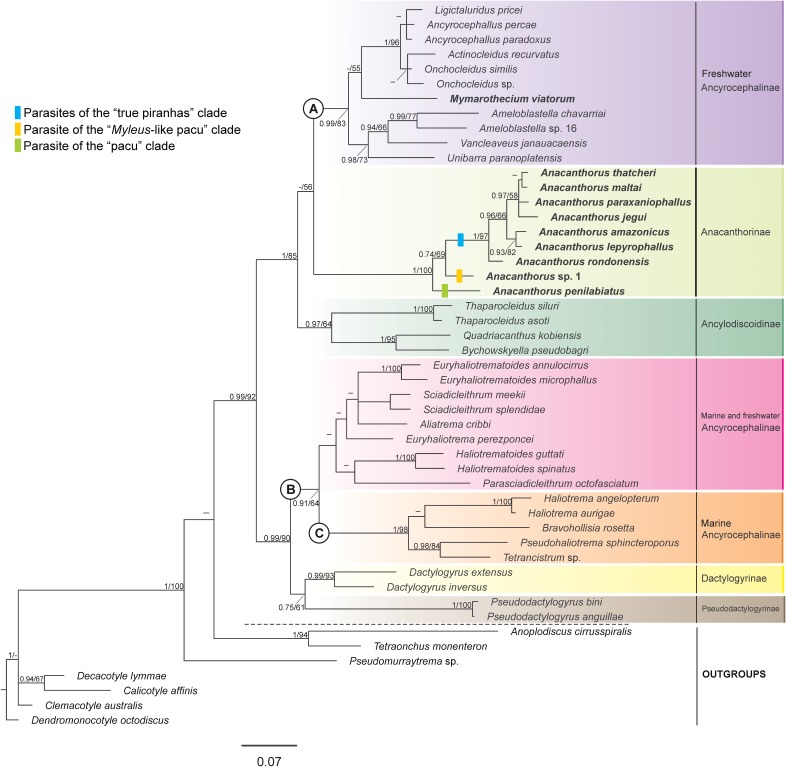
Consensus Bayesian topology from the phylogenetic analysis of partial 28S rDNA of 49 species of monogeneans. BI posterior probabilities and ML bootstrap values are shown at the nodes. Posterior probabilities <0.7 are not reported. Bootstrap values <50 are not reported. Species sequenced in the present study are shown in bold.

New partial 28S rDNA sequences were obtained for nine species of *Anacanthorus* (*Anacanthorus amazonicus* Van Every & Kritsky, 1992 [65], *Anacanthorus jegui* Van Every & Kritsky, 1992 [65], *Anacanthorus lepyrophallus* Kritsky, Boeger, and Van Every, 1992 [29], *Anacanthorus maltai* Boeger & Kritsky, 1988 [5], *Anacanthorus paraxaniophallus* Moreira, Carneiro, Ruz & Luque, 2019 [42], *Anacanthorus penilabiatus* Boeger, Husak & Martins, 1995 [6], *Anacanthorus rondonensis* Boeger & Kritsky, 1988 [5], *Anacanthorus thatcheri* Boeger & Kritsky, 1988 [5] and *Anacanthorus* sp. 1) and *Mymarothecium viatorum*, and varied from 612 bp to 716 bp (when using the C1 and D2 primers) and from 1425 bp to 1434 bp (when using the U178 and L1642 primers). Specimens identified as *Anacanthorus* sp. 1 represented an undescribed species parasitizing *Myleus setiger*. An unambiguous alignment of all analyzed species of the Dactylogyridea and Monocotylidea spanned 391 positions and included 205 parsimony-informative characters, 227 variable characters, and 164 conserved characters. ML and BI analyses generated phylogenetic trees with similar general topology and the monophyly of *Anacanthorus* was strongly supported by both analyses (Fig. 1). The Anacanthorinae, represented only by *Anacanthorus* spp. in this study, appeared to form a monophyletic group clustering with clade A comprising solely freshwater species of Ancyrocephalinae and the clade of *Ancylodiscoidinae* spp. *Anacanthorus penilabiatus* showed the basal position within the clade of *Anacanthorus* spp. Even though the ML and BI phylogenetic trees displayed the same topology, the status of *Anacanthorus* as a sister group to clade A of freshwater Ancyrocephalinae was only weakly supported by ML analysis. *Mymarothecium viatorum*, the next host-specific monogenean representative parasitizing serrasalmids, was positioned within clade A of the Ancyrocephalinae.

Clade B, including representatives of both freshwater and marine species of the Ancyrocephalinae, was well supported by BI analysis and weakly supported by ML analysis. The Dactylogyrinae formed a monophyletic sister group to Pseudodactylogyrinae (only weakly supported) and clustered with clade B of Ancyrocephalinae. Within clade B of Ancyrocephalinae, five marine species formed a well-supported group (clade C) on the basis of both analyses.

Within the Anacanthorinae i.e., *Anacanthorus*, the phylogenetic relationships among *Anacanthorus* seemed to reflect the phylogeny of their serrasalmid hosts. *Anacanthorus penilabiatus* from *Piaractus mesopotamicus*, a member of the “pacu” clade, showed the basal position within *Anacanthorus*; the following position was held by *Anacanthorus* sp. 1 from *Myleus setiger* (this position was weakly or moderately supported by BI and ML analyses, respectively), a representative of the “*Myleus*-like pacus” clade. Finally, the large group of *Anacanthorus* included two clades of species from hosts representing the “true piranhas” lineage, the first one well supported and including *A. lepyrophallus* and *A. amazonicus*, the second one including *A. paraxaniophallus*, *A. jegui*, *A. thatcheri* and *A. maltai*. *A. rondonensis* from *Pygocentrus nattereri*, a representative of the “true piranhas” lineage, showed the basal position in this large *Anacanthorus* group.

## Discussion

In the present study, and for the first time, the phylogenetic position of *Anacanthorus* within the Dactylogyridae was evaluated on the basis of analyses of partial 28S rDNA sequences. Representatives of five subfamilies within the Dactylogyridae, i.e., Ancyrocephalinae, Ancylodiscoidinae, Anacanthorinae, Dactylogyrinae, and Pseudodactylogyrinae, were included in the analyses. Using molecular data, we confirmed the monophyly of the Anacanthorinae (here represented only by *Anacanthorus*), in accordance with previous studies based on morphological characters [28, 65]. We did not include any member of *Anacanthoroides* in the phylogenetic analyses.

Our results show that phylogenetic patterns between *Anacanthorus* spp. correspond to those between the Serrasalmidae. Ortí et al. [47] inferred the first molecular phylogeny of Serrasalmidae using mtDNA (12S and 16S rRNA) markers, and found three major lineages, (i) a clade including the “pacus” in the most basal position, followed by (ii) the clade including “*Myleus*-like pacus” species, and (iii) a clade including the most diverse group of Serrasalmidae, represented by the “true piranhas”. They also determined the placement of *Acnodon* as a sister group to the two last lineages and suggested the paraphyly of some genera, i.e., *Myleus*, *Pristobrycon* and *Serrasalmus*. Later, Ortí et al. [48] performed analyses based on complete sequences of the mtDNA control region (D-loop) and on partial sequences of 12S and 16S rRNA, and their findings corroborated the previous division into three main lineages; they also suggested that other serrasalmid genera are not monophyletic. Finally, more recently, Thompson et al. [64] performed a robust phylogenetic analysis based on the sequences of 10 nuclear genes (two exons and eight introns) and the mtDNA control region. Their results agreed with previous studies on the phylogenetic relationships within serrasalmids based on mtDNA and confirmed that there are still many gaps to fill with regard to the taxonomy of this fish group.

Our results may suggest that cospeciation processes played a role between *Anacanthorus* spp. and their serrasalmid hosts (at least at the level of three serrasalmid lineages). Recently, Graça et al. [20] suggested that there is cospeciation between *Anacanthorus* and their host lineages representing different families (Serrasalmidae, Bryconidae and Erythrinidae), even though duplications were the most frequent coevolutionary event in the speciation of *Anacanthorus* parasitizing species of the same family. In fact, cospeciation between monogeneans and their hosts was not found to be significant in some extensively studied groups such as *Lamellodiscus* [11], *Gyrodactylus* [74], *Dactylogyrus* [58, 59] and *Cichlidogyrus* [37].

The phylogenetic relationships among *Anacanthorus* spp. also seem to reflect the similarity in the morphology of the copulatory complex. Although we did not analyze all species previously morphologically evaluated by Van Every and Kritsky [65], their phylogenetic reconstruction using the morphology of copulatory complex is similar to our phylogenetic reconstruction using molecular data (i.e., the species analyzed in both studies exhibited the same phylogenetic relationships). However, to effectively investigate the congruence of phylogenies built on molecular and morphological data, the sequencing of a larger dataset of *Anacanthorus* species is necessary in future studies, potentially focusing on mapping the characters of the copulatory complex into the molecular phylogenetic reconstruction.

According to our results, species of *Anacanthorus* formed a clade including the group of freshwater members of the Ancyrocephalinae (clade A) and the group of species of Ancylodiscoidinae. At the same time, we showed that *Mymarothecium viatorum*, an exclusive parasite of the “pacu” lineage, was positioned within freshwater Ancyrocephalinae. Using complete 18S rDNA sequences, Müller et al. [45] showed that *M. viatorum* clustered with *A. penilabiatus*. Both ribosomal markers (28S and 18S rDNA) have been widely used to reconstruct the phylogenies of monogeneans, and in many cases they have produced similar topologies (e.g., Plaisance et al. [51], Francová et al. [15], Verma et al. [66]); thus, the finding of Müller et al. [45] is due to the lack of sequences of species closely related to *Anacanthorus* species (i.e., the absence of the representatives of freshwater Ancyrocephalinae).

We conclude that *Anacanthorus* and their serrasalmid hosts can provide a useful model for studying host-parasite biogeography and coevolution in the neotropics. However, to perform cophylogenetic analyses, future studies are needed focusing on a wider spectrum of host species and their specific *Anacanthorus* spp. Additional sampling of the representatives of other monogenean genera parasitizing serrasalmids will allow us to investigate the phylogenetic relationships among such diverse monogeneans parasitizing the same host group.
